# Granulocyte colony-stimulating factor-associated aortitis in a woman with breast cancer: a case report

**DOI:** 10.1186/s40792-022-01514-6

**Published:** 2022-08-18

**Authors:** Nana Matsumoto, Naoto Kondo, Yumi Wanifuchi-Endo, Tomoko Asano, Tomoka Hisada, Yasuaki Uemoto, Akiko Kato, Mitsuo Terada, Natsumi Yamanaka, Ayaka Isogai, Muneyuki Takayama, Takeshi Hasegawa, Koichi Ito, Keiji Mashita, Tatsuya Toyama

**Affiliations:** 1 Department of Surgery, Inazawa Kosei Hospital, 7 Zichono, Sobuechohonko, Inazawa 495-8531 Japan; 2 Department of Breast Surgery, Nagoya City University Graduate School of Medical Sciences, 1 Kawasumi, Mizuho-cho, Mizuho-ku, Nagoya, 467-8601 Japan

**Keywords:** Granulocyte colony-stimulating factor (G-CSF), Aortitis, Breast cancer, Pegfilgrastim

## Abstract

**Background:**

Granulocyte colony-stimulating factor (G-CSF) is increasingly used to prevent chemotherapy-associated febrile neutropenia. Generally, aortitis is not considered a side effect of G-CSF and is thought to be extremely rare. Aortitis is an inflammation of the aorta and occurs mainly in connective tissue diseases (Takayasu arteritis, giant cell arteritis, etc.) and infectious diseases (bacterial endocarditis, syphilis, etc.). We report herein a rare case of G-CSF associated with aortitis in a woman with breast cancer.

**Case presentation:**

Here, we present a case involving a 63-year-old woman with luminal type stage IIa breast cancer. The patient’s treatment was initiated with docetaxel and cyclophosphamide, with pegfilgrastim (PEG-G) as support. After PEG-G administration on day 3, the patient developed an intermittent fever of up to 39.4 °C on day 10 and visited our outpatient clinic on day 13 with persistent high fever. Laboratory tests revealed a high neutrophil count (14,000/μL) and a high C-reactive protein (CRP) level (42.8 mg/dL) without any other abnormalities. Contrast-enhanced computed tomography scanning revealed soft tissue thickening with weak enhancement around the wall of the thoraco-abdominal aorta, aortic arch and left subclavian artery. The patient did not respond to antimicrobial agents. On the basis of these observations, the patient was diagnosed with PEG-G-induced aortitis, and her condition rapidly improved without corticosteroids.

**Conclusions:**

Clinicians should be aware of aortitis as a potential complication in patients undergoing G-CSF chemotherapy. In cases with persistent high fever after PEG-G administration, and in the absence of infection, aortitis should be suspected.

## Background

Pegfilgrastim (PEG-G) is a long-acting granulocyte colony-stimulating factor **(**G-CSF**)** widely used to prevent chemotherapy-associated febrile neutropenia in cancer patients. G-CSF preparations bind to G-CSF receptors on neutrophil progenitor cells in the bone marrow, promoting their differentiation into neutrophils. The most common adverse effects of G-CSF are bone pain and injection-site reactions, although generally less serious adverse events have been reported [1]. According to the Japanese Adverse Drug Event Report (JADER) by the Pharmaceuticals and Medical Devices Agency (PMDA), aortitis is an adverse effect of G-CSF, although this has rarely been reported [2]. Here, we report a rare case of PEG-G-induced aortic inflammation in a patient receiving post-operative chemotherapy for early breast cancer.

## Case presentation

The case involved a 62-year-old postmenopausal woman without any personal or family medical history. The patient underwent a left mastectomy and sentinel lymph node biopsy for left breast cancer. A pathological examination revealed the following: invasive ductal carcinoma, pT2 (22 mm), nuclear grade 3, pN0, estrogen receptor (ER) and progesterone receptor (PgR) positive, human epidermal growth factor receptor 2 (HER2) negative, and pT2N0M0 stage IIa.

On day 1, the patient was administered a chemotherapy regimen consisting of docetaxel (75 mg/m^2^) and cyclophosphamide (600 mg/m^2^), together with dexamethasone (6.8 mg). The patient was administered oral dexamethasone (8 mg) on days 2–4 and subcutaneous PEG-G on day 3. On day 10 (day 8 after PEG-G administration), the patient complained of intermittent high fever (up to 39.4 °C); the fever persisted even after the administration of levofloxacin (LVFX), which was prescribed for febrile neutropenia. In addition to fever, the patient developed other symptoms, including loss of appetite and shortness of breath, and visited our hospital on day 13 (11 days after PEG-G administration). At admission, the patient’s body temperature was 39.2 °C. Laboratory examination revealed a high neutrophil count (14,000/μL) and elevated CRP (42.78 mg/dL) without any other abnormalities. The erythrocyte sedimentation rate was 76 mm/h. The levels of anti-nuclear antibody (ANA), myeloperoxidase anti-neutrophil cytoplasmic antibody (MPO-ANCA), serine proteinase 3 anti-neutrophil cytoplasmic antibody (PR3-ANCA), and rheumatoid factor were within normal limits. To rule out viral infections, we performed an antibody test for human T-cell lymphotropic virus type 1, cytomegalovirus, and human parvovirus B 19, which were all negative. No bacterial growth was observed in blood and urine cultures (Table 1).

**Table 1:** Results from laboratory examination on admission

Blood cell count		Normal values
White blood cells (/µL)	15,600	3000–9000
Neutrophils (%)	90.0	40.0–69.0
Eosinophils (%)	0	0–5.0
Basophil (%)	6.0	0–2.0
Lymphocytes (%)	3.0	26.0–46.0
Red blood cells (/µL)	3.54 × 10^6^	3.53 × 10^6^–5.25 × 10^6^
Hemoglobin (g/dL)	10.5	10.6–16.5
Platelet count (/µL)	22.5 × 10^3^	13.8 × 10^3^–30.9 × 10^3^
CRP (mg/dL)	42.8	≦ 0.3

**Fig. 1:**
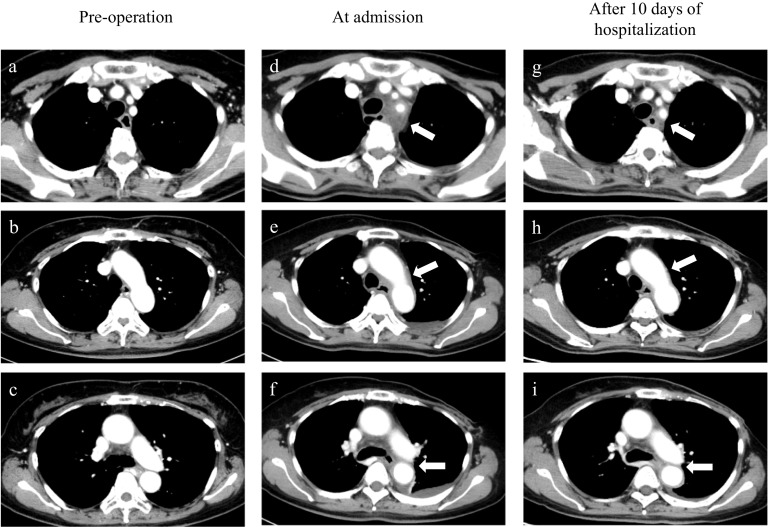
Pre-operation computed tomography (CT) revealed no abnormalities around the aorta (**a**–**c**). CT scanning at the time of admission (**d**–**f**) revealed thickening of the arterial wall of the thoracic and abdominal aorta and increased fat concentration in the surrounding area. A CT scan after 10 days of hospitalization revealed reduced arterial thickening and fat concentration relative to the CT at the time of admission (**g**–**i**)

Contrast-enhanced computed tomography (CT) scan on pre-operation showed no abnormalities around the aorta (Fig. 1a–c). At the time of admission, CT scanning revealed soft tissue thickening with weak enhancement around the wall of the thoraco-abdominal aorta, aortic arch and left subclavian artery (Fig. 1d–f). From the patient’s clinical course and imaging data, we suspected G-CSF-associated aortitis. At that point, we could not rule out infection because we had no results of autoantibodies, viral antibody tests, or blood or urine cultures, so we continued antibiotics. On day 8 after admission, the patient’s fever resolved spontaneously and the neutrophil count and CRP level decreased to 3185/μL and 17.88 mg/dL, respectively. Based on the results of the autoantibody test, viral antibody test, and blood and urine culture tests at admission, the patient was considered not to have an infection, and antibiotics were discontinued. Corticosteroids were not administered because of the patient’s general improvement. 10 days after admission (20 days after PEG-G administration). The thickening around the aorta was reduced on CT scanning (Fig. 1g, h, Table 2).

**Table 2:** Timeline of patient treatment

Day 1	Chemotherapy (docetaxel 75 mg/m^2^ and cyclophosphamide 600 mg/m^2^) was administered along with dexamethasone (6.8 mg)
Days 2–4	Dexamethasone (8 mg) was administered orally
Day 3	PEG-G was administered
Day 10	The patient developed a high fever and treatment with LVFX was started
Day 13	The patient was hospitalized with persistent fever, poor oral intake, and shortness of breath
Day 20	The fever resolved and the neutrophil and CRP levels fell. LVFX was discontinued
Day 22	The thickening around the aorta was reduced on CT scanning

Chemotherapy was then discontinued and hormone therapy started. No recurrence of aortitis was observed for 15 months.

## Discussion

Aortitis is classified as either non-infectious or infectious. Although the mechanisms of aortitis are not fully understood, non-infectious aortitis might be triggered by increased neutrophil-mediated damage and pro-inflammatory reactions (3). G-CSF stimulates proliferation and differentiation of neutrophil precursors [4] and the resulting immunological reactions can cause aortitis [5].

In the case reported here, we excluded autoimmune disease because all ANA, MPO-ANCA, and PR3-ANCA values were within normal ranges. Since viral antibody tests were also negative and no bacterial growth was observed in blood and urine cultures, we concluded that G-CSF was the most suspicious cause of this aortitis.

Corticosteroids are commonly used to treat patients with suspected autoimmune diseases, such as Takayasu arteritis (TAK). However, there is no established treatment for G-CSF-associated aortitis. In general, G-CSF-associated aortitis has good prognosis and often resolves spontaneously without the administration of corticosteroids [6]. Moreover, previous reports show that there is no difference in the therapeutic effect and the time to remission of G-CSF-associated aortitis with or without corticosteroids [7]. The cases of G-CSF-associated aortitis reported in recent years are listed in Table 3 [6–13].

**Table 3:** Reported **cases of G-CSF-associated aortitis in recent years**

References	Age G-CSF	Period^a^	Location of aortitis	Steroid treatment	Re-administration of G-CSF
[6]	65 PEG-G	8–18	Aortic arch	None	Re-administered and **aortitis** recurred
[7]	72 PEG —G	4–14	Thoracic	None	None
[8]	52 Details unknown	14–38	Aortic arch and abdominal	Prednisolone 50 mg	None
[9]	58 PEG -G	8–21	Right subclavian	None	None
[10]	43 PEG-G	8–36	Aortic arch	Prednisolone 60 mg	None
[11]	77 Details unknown	8–21	Bilateral common carotid and left subclavian	None	None
[12]	61 PEG-G	7–17	Thoracic	None	None
[13]	60 Details unknown	5–15	Thoraco-abdominal	Prednisolone 60 mg	None
[13]	70 Filgrastim	15–25	Thoraco-abdominal	Prednisolone 60 mg	None
Present case	62 PEG -G	7–17	Thoraco-abdominal, aortic arch and left subclavian	None	None

PEG-G: pegfilgrastim

^a^Days with fever from G-CSF administration

Aortitis caused by PEG-G tended to develop within 2 weeks of G-CSF administration and to resolve spontaneously within 3 weeks, as in the present case. We hypothesized that this common period of time was due to the duration of action of PEG-G, and that once the effects of PEG-G wore off, the aortitis would spontaneously abate. What is also interesting about this case is that despite the extensive lesions, the disease resolved quickly and spontaneously without corticosteroid treatment. This suggests that the extent of the lesions is not related to the need for corticosteroid treatment. On the other hand, it has been reported that G-CSF-associated aortitis lasting more than 3 weeks was relieved by corticosteroid administration, and we believe that corticosteroids should be considered in cases of long-term lack of improvement.

Although there are reports of G-CSF re-administration after symptom improvement in patients with G-CSF-associated aortitis, caution is required because some patients had recurrent aortitis [6, 14]. In this case, at the patient’s request, we shifted to endocrine therapy without re-administering PEG-G, and aortitis recurrence was not observed for 15 months after the onset of symptoms.

In breast cancer patients, dose-dense chemotherapy involving more frequent administration of chemotherapy agents than in standard chemotherapy significantly improved clinical outcomes [15, 16]. The addition of G-CSF to chemotherapy regimens to prevent chemotherapy-induced febrile neutropenia yields favorable clinical outcomes in breast cancer patients [17, 18]. Therefore, physicians should be aware that because most breast cancer patients undergo chemotherapy with PEG-G, aortitis may easily develop.

## Conclusion

Clinicians should be aware of aortitis as a possible complication in patients undergoing chemotherapy with G-CSF. In cases with persistently high fever after G-CSF administration, aortitis should be suspected and a thoraco-abdominal CT scan or scintigraphy should be performed at an early stage.

## Data Availability

All data underlying this report are included within the article.

## Abbreviations

G-CSF: Granulocyte colony-stimulating factor
PEG-G: Pegfilgrastim
CRP: C-reactive protein
JADER: Japanese Adverse Drug Event Report
PMDA: Pharmaceuticals and Medical Devices Agency
ER: Estrogen receptor
PgR: Progesterone receptor
HER2: Human epidermal growth factor receptor 2
LVFX: Levofloxacin
ANA: Anti-nuclear antibody
MPO-ANCA: Myeloperoxidase anti-neutrophil cytoplasmic antibody
PR3-ANCA: Serine proteinase 3 anti-neutrophil cytoplasmic antibody
TAK: Takayasu arteritis
